# Shockwaves delivery for aortic valve therapy—Realistic perspective for clinical translation?

**DOI:** 10.3389/fcvm.2023.1160833

**Published:** 2023-04-11

**Authors:** Lavinia Curini, Maurizio Pesce

**Affiliations:** Unità di Ricerca in Ingegneria Tissutale Cardiovascolare, Centro Cardiologico Monzino, IRCCS, Milan, Italy

**Keywords:** calcific aortic valve disease, ultrasound, lithotripsy, decalcification, medical device

## Abstract

Calcific aortic valve disease (CAVD) is the most frequent valvular heart disorder, and the one with the highest impact and burden in the elderly population. While the quality and standardization of the current aortic valve replacements has reached unprecedented levels with the commercialization of minimally-invasive implants and the design of procedures for valve repair, the need of supplementary therapies able to block or retard the course of the pathology before patients need the intervention is still awaited. In this contribution, we will discuss the emerging opportunity to set up devices to mechanically rupture the calcium deposits accumulating in the aortic valve and restore, at least in part, the pliability and the mechanical function of the calcified leaflets. Starting from the evidences gained by mechanical decalcification of coronary arteries in interventional cardiology procedures, a practice already in the clinical setting, we will discuss the advantages and the potential drawbacks of valve lithotripsy devices and their potential applicability in the clinical scenario.

## 1. The role of cells-depositing calcium in CAVD

Calcific aortic valve disease (CAVD) is the most frequent heart valve disorder in the aging population, associated with increased morbidity and mortality (1). The underlying process of CAVD is typically illustrated by a complex and multifaceted course, characterized by endothelial dysfunction, inflammation, increased oxidative stress, sub-endothelial lipid accumulation, valve fibrosis and, ultimately, calcification of valve leaflets (2). The clinical evolution of the valve pathology starts with aortic sclerosis, characterized by mild valve thickening; it evolves into symptomatic aortic stenosis (AS), characterized by obstruction of blood flow, severe calcification preventing leaflet movement, and heart failure (3, 4). This process occurs in a typically biphasic fashion. The sclerotic phase is slow with mild or no symptoms, followed by severe calcification with dyspnea, angina and myocardial decompensation (5).

Mechanistically, the deposition of calcific nodules in the aortic valve generally begins in the fibrosa, a layer that is predominantly abundant of type I and III collagen forming anisotropically-deposited thick fibers necessary to absorb the load generated by the blood filling the aorta during the valve closure at diastole (6). This establishes a relationship between the non-uniform distribution of strain forces on the leaflets and the pathologic programming of valve-resident cells.

Calcific lesions are mainly produced from valve-resident cells, whose the most important type are the so-called valve interstitial cells (VICs) (7). These cells are normally deputed to the renewal of the extracellular matrix, but under specific pathophysiologic conditions, they can differentiate into myofibroblasts (8) and finally into calcium depositing cells (9), under the control of genes (e.g., Bmp2, Runx2), at least in part, in common with the canonical osteogenic pathway. Despite the similarity with the bone calcification process, valve and vessel-specific mineralized tissues show a different organization. Indeed, in an interesting work, Bertazzo and colleagues, using nano-analytical electron microscopy techniques, detected spherical calcium phosphate particles, made of highly crystalline hydroxyapatite and structurally different from mineralized bone (10). They showed that, unlike tissue presenting calcific lesions that clearly express bone-specific factors, the deposition of spherical microparticles in the extracellular matrix precedes the accumulation of the large calcific nodules present in the pathologic valves (10). Interestingly, the same Authors showed that deposition of these particles was more abundant in the fibrosa layer in positions subjected to the maximal mechanical stress (11). These evidences, together with experiments *in vitro* showing that secretion of calcified particles by VICs is subjected to mechanical control (12), and that these cells are sensitive to mechanical cues (13), confirm the primary relevance of valve mechanics for pathologic evolution, and suggest that removing the calcium deposits by a debridement technique, could be a viable strategy to recover a normal phenotype in valve resident cells, other than restoring the mechanical function of the valve.

Other cell types that contribute to calcific evolution of the aortic valves are the endothelial cells that cover the leaflet surface, the so-called valve endothelial cells (VECs), which similarly to the VICs can participate in the calcification of the valve by differentiating into mesenchymal cells through endothelial-mesenchymal transition (14). Several conditions such as altered shear stress, inflammation and modifications in the extracellular matrix (15–17) can favor VECs differentiation into myofibroblasts and, subsequently into calcium-depositing cells.

A last relevant cell type participating in valve calcification has been recently highlighted by the finding that somatic blood cell-derived clones bearing somatic mutations in *DNMT3A* or *TET2* loci predominate in the peripheral blood of patients with an increased mortality rate following TAVI implantation. This expansion, named “clonal hematopoiesis of indeterminate potential” (CHIP) is supposed to create a proinflammatory environment characterized by increased pro-inflammatory leukocyte subsets and a pro-inflammatory T-cell polarization likely favoring rapid progression of aortic stenosis and its complications (18–20).

## 2. Use of shockwaves in treatment of cardiovascular diseases

### 2.1. Intravascular lithotripsy

The term lithotripsy refers originally to a technique that employs sonic pressure waves—or shockwaves—to disintegrate and remove hard deposits such as renal and ureteral calculi or gallstones, whose remnants are later washed out by urinary or biliary secretion (21–23). Shockwaves are in use also in cardiovascular therapy. However, opposite to the original destination of use, the effect of the mechanical treatment is not that of removing the calcium from the tissues, but to reduce the size of the deposits with the aim at facilitating interventional cardiology procedures in heavily calcified coronary arteries (24–31), or to create easier vascular access for minimally-invasive procedures in case of calcifications in the iliac arteries (32–34). Given that the final aim of the treatment is to restore the softness of the tissue, in these applications, the calcium deposits remain *in situ* and are not expected to be washed-out by the blood giving rise to thromboembolic events. The delivery of shockwaves to the vessels is named “intravascular lithotripsy” (IVL). It is performed exploiting the minimally-invasive procedure and setup available in the interventional cardiology room contextually with the revascularization (35). Technically, the shockwaves are generated by piezoelectric lithotripters inserted into balloon catheters connected to a generator producing adjustable doses and intensities to optimize the treatment of the arteries for each specific patient (25). The sonic waves are transmitted from the balloon-based catheter to the vascular wall by physical contact to reach the media of the vessels, where they break more superficial or deeper calcium deposits, allowing optimal artery expansion and stent implantation. In the clinical practice, IVL has been successfully employed in balloon angioplasty and plaque ablation and as an adjuvant to prevent post-procedure complications, such as accelerated restenosis, damage of the arterial wall, or catheter overstretching and rupture (36–39). In a trial by Tepe et al., the authors tested the efficiency of IVL on percutaneous transluminal angioplasty (PTA) in two cohorts of patients with femoropopliteal artery calcification (40). Data reported a greater success in the patient group who received IVL before PTA compared to the PTA-only group, thus confirming IVL as an effective vessel preparation method facilitating endovascular treatment (40). In another trial, Hill and colleagues showed the safety and effectiveness of IVL to allow stent implantation in 431 patients with calcified coronary lesions (30). The percentage of procedural success was 92.4%, with no adverse events (e.g., myocardial infarction, cardiac death). The IVL safety was also evaluated in stents under-expansion and in-stent restenosis in 60 patients who underwent percutaneous coronary intervention with intravascular lithotripsy system for severe calcified lesions (41). This analysis showed that the IVL balloon easily reached the lesion, and the application of the treatment was feasible in 92.3% of cases, with high angiographic success and no differences in complications or major cardiac adverse events at 30-days, confirming IVL as a safe strategy to adjuvate stent expansion (41, 42).

### 2.2. Shockwave treatment in minimally-invasive aortic valve replacement

Similarly to the use in coronary revascularization, setups for IVL have been successfully employed to facilitate the intravascular access to the aortic valve for easier insertion of large-dimension sheaths in TAVI procedures (43–46). Shockwaves are delivered by a piezoelectric device to the iliac artery or the aortic wall to break up the calcifications hardening the tissue, allowing the insertion of the catheters for TAVI implantation and reducing the risks of arterial wall damage to optimize TAVI deployment (46). For example, in two studies in patients aged 75–89 years with AS and presenting more than one lesion that required pre-procedural intervention, IVL simplified the femoral access and TAVI implantation (47, 48). Another use of lithotripsy for facilitating valve replacement was described by Sharma and colleagues, who performed transcatheter aortic valve lithotripsy directly in the valve before TAVI implantation to decrease the risk of paravalvular aortic regurgitation and annular rupture. The results showed that the procedure did not affect the motion of the leaflets in the prosthetic valve and favored the expansion of the TAVI stent (49). Similarly, a post TAVI dilatation lithotripsy was performed in a patient carrying a previously implanted TAVI to allow a new expansion of the TAVI stent in order to reduce the risk of stroke or annular rupture (50). Results showed an effectively post-dilated valve and a more symmetric expansion of the supporting stent.

### 2.3. Shockwaves delivery for direct calcium disruption in human calcified leaflets

A new application of shockwaves delivery, as an adjuvant or even a stand-alone treatment, for aortic valve disease concerns the disintegration of the calcific deposits reducing the pliability and the motion of the leaflets in terminally calcified stenotic valves. This interesting possibility was prospected in a report from our group (51), in which we described the feasibility of treating aortic valve leaflets using an *ad-hoc* device that was specifically designed to deliver shockwaves in a very localized and concentrated fashion, by direct physical contact with the leaflet of a tricuspid valve. Conceived to be part of an all-in one “trans-catheter debridement device” (TDD), to treat the valves with low-intensity ultrasound shockwaves with alternate 100 kHz/3 MHz pulses with a minimally-invasive trans-catheter approach, we showed the efficiency of the emitted waves in reducing the dimensions of the calcific nodules in pathologic human leaflets *ex vivo*, and the safety of the shockwaves administration to the aortic valves in living pigs (51). The absence of major histologically detectable damages witnessed that for their extreme focalization to penetrate the large calcium deposits from the aortic leaflets, the shockwaves did not cause large ruptures maintaining the integrity of the tissue (51, 52). For the whole duration of the procedure, the animals remained with one of the valve leaflets immobilized by the piezoelectric transducer, even if the motion of the other leaflets during the procedure, as well as of the treated leaflet after the procedure were not compromised, suggesting an overall clinical feasibility of the procedure. The biological safety of the procedure was finally confirmed in another report in which the same device was employed to treat an *in vitro* reconstituted valve tissue, where no major ruptures and no changes in cell viability were observed (53). At the moment, it is not known whether the delivery of shockwaves to the leaflets results into an induction of inflammation and/or cellular apoptosis (54) and the permanence of smaller deposits resulting from the fragmentation of the large calcific nodules causes long-term effects on mechanical performance of the valve. Another important aspect of this potentially new treatment will probably be the necessity to employ, concomitantly to shockwaves delivery, embolic protection devices able to filter out from the blood the debris deriving from the calcium deposits fragmentation. In this respect, several types of these devices have been designed to capture debris that embolizes distally during vascular surgeries in specific vascular districts particularly critical such as, for example, carotid stenting (55).

## 3. Conclusion

The growing interest for minimally-invasive procedures to reduce the extent of calcification in vessels and valves, and to increase the efficiency of vessel reperfusion and valve substitution, is prompting the design of a new class of devices that will be employed in the future to minimize the clinical consequences of cardiovascular aging. Prompted by pioneering studies on isolated cases or small trials, delivery of shockwaves is progressing in preclinical testing in preparation for possible large human translation. Before the release of a regulatory-compliant procedure to debride the large calcific nodules present in the natural valves, or even in implanted biological valves, this technology should be validated for safety in terms of long-term biological effects and should be designed according to quality criteria. In addition, the risks should be minimized, for example, by combining the new shockwaves delivery system with distal protection devices able to collect eventual debris originating from the calcium disintegration activity.
